# Systemic Inflammation, Nutritional Status and Tumor Immune Microenvironment Determine Outcome of Resected Non-Small Cell Lung Cancer

**DOI:** 10.1371/journal.pone.0106914

**Published:** 2014-09-19

**Authors:** Marco Alifano, Audrey Mansuet-Lupo, Filippo Lococo, Nicolas Roche, Antonio Bobbio, Emelyne Canny, Olivier Schussler, Hervé Dermine, Jean-François Régnard, Barbara Burroni, Jérémy Goc, Jérôme Biton, Hanane Ouakrim, Isabelle Cremer, Marie-Caroline Dieu-Nosjean, Diane Damotte

**Affiliations:** 1 Deparment of Thoracic Surgery, Paris Centre University Hospitals, AP-HP, Paris, France; 2 University Paris Descartes; Paris, France; 3 Deparments of Pathology, Paris Centre University Hospitals, AP-HP, Paris, France; 4 INSERM U1138, Cancer and Immune Escape, Cordeliers Research Center, Paris, France; 5 University Pierre and Marie Curie, UMRS U1138, Paris, France; 6 Unit of thoracic Surgery, IRCCS-Arcispedale Santa Maria Nuova, Reggio Emilia, Italy; 7 Departments of Chest Disease, Paris Centre University Hospitals, AP-HP, Paris, France; Memorial Sloan-Kettering Cancer Center, United States of America

## Abstract

**Background:**

Hypothesizing that nutritional status, systemic inflammation and tumoral immune microenvironment play a role as determinants of lung cancer evolution, the purpose of this study was to assess their respective impact on long-term survival in resected non-small cell lung cancers (NSCLC).

**Methods and Findings:**

Clinical, pathological and laboratory data of 303 patients surgically treated for NSCLC were retrospectively analyzed. C-reactive protein (CRP) and prealbumin levels were recorded, and tumoral infiltration by CD8+ lymphocytes and mature dendritic cells was assessed. We observed that factors related to nutritional status, systemic inflammation and tumoral immune microenvironment were correlated; significant correlations were also found between these factors and other relevant clinical-pathological parameters. With respect to outcome, at univariate analysis we found statistically significant associations between survival and the following variables: Karnofsky index, American Society of Anesthesiologists (ASA) class, CRP levels, prealbumin concentrations, extent of resection, pathologic stage, pT and pN parameters, presence of vascular emboli, and tumoral infiltration by either CD8+ lymphocytes or mature dendritic cells and, among adenocarcinoma type, tumor grade (all p<0.05). In multivariate analysis, prealbumin levels (Relative Risk (RR): 0.34 [0.16–0.73], p = 0.0056), CD8+ cell count in tumor tissue (RR = 0.37 [0.16–0.83], p = 0.0162), and disease stage (RR 1.73 [1.03–2.89]; 2.99[1.07–8.37], p = 0.0374- stage I vs II vs III-IV) were independent prognostic markers. When taken together, parameters related to systemic inflammation, nutrition and tumoral immune microenvironment allowed robust prognostic discrimination; indeed patients with undetectable CRP, high (>285 mg/L) prealbumin levels and high (>96/mm2) CD8+ cell count had a 5-year survival rate of 80% [60.9–91.1] as compared to 18% [7.9–35.6] in patients with an opposite pattern of values. When stages I-II were considered alone, the prognostic significance of these factors was even more pronounced.

**Conclusions:**

Our data show that nutrition, systemic inflammation and tumoral immune contexture are prognostic determinants that, taken together, may predict outcome.

## Introduction

Lung carcinoma is a leading cause of cancer-related death worldwide [1]–[2]. Despite progresses in chemotherapy and biologically-targeted therapy and refinement in multimodal therapeutic combinations, long-term outcome remains poor, with the exception of stage IA disease, stressing the need for research to better understand the biology of the disease and factors conditioning long-term survival and risk of relapse [3]–[6].

The interactions between systemic inflammation and tumoral immune microenvironment are increasingly investigated in cancer patients [7]–[9]. Pro-inflammatory cytokines and associated growth factors are involved in carcinogenesis through their effects on tumor cell growth, survival, proliferation and migration [10]. It has been shown that slight elevations of inflammatory markers are associated with an increased risk of non-small cell lung carcinoma (NSCLC) occurrence [11]–[12], and serum C-reactive protein (CRP) has been identified as a prognostic factor in both advanced and resectable NSCLC [13], [14]. The tumoral immune microenvironment has been also shown to be an important determinant of long-term outcome in primary and metastatic tumors [9]: particularly in NSCLC, high levels of mature dendritic cells (mDC) and of CD8+ lymphocytes have been both identified as robust prognostic factors [15]–[17].

Lung cancer is frequently associated with chronic bronchitis and chronic obstructive pulmonary disease (COPD) [1]. COPD is also associated with systemic inflammation and shares several risk factors and pathophysiological mechanisms with NSCLC, including airways inflammation, protease/antiprotease imbalance, oxidative stress and abnormal repair mechanisms [18]. In COPD, markers of systemic inflammation are related to survival and nutritional status [18]. Nutritional status is also a determinant of survival in lung cancer patients, as illustrated by the association between prognosis and low albumin levels [14], [19] or low body mass index (BMI) [19]. Low pre-albumin levels have also been found to be associated to early recurrence and poorer short-term outcome in resected NSCLC [14], [20].

Nowadays, no study have been performed to investigate the respective impact of systemic inflammation, nutritional status, and immune microenvironment on the survival of patients with resected NSCLC and to assess the interactions between these factors and the outcome. The present study was designed to address these issues in a large series of patients with resected NSCLC.

## Patients and Methods

Clinical and pathological data of 303 consecutive patients who underwent major lung resection for NSCLC at Hôtel-Dieu University Hospital in Paris between June 2001 and December 2002 were retrospectively analysed. The research was conducted according to recommendations outlined in the Helsinki declaration. IRB approval was obtained (Comité de Protection des Personnes [CPP] Ile de France II, n° 2008-133 and 2012 06-12). IRB dispensed from obtaining informed consent, because of the retrospective, non-interventional character of the study and the high number of deceased patients when the study was performed. Patient records were anonymized and de-identified prior to analysis.

### Inclusion/Exclusion Criteria

Patients were not included if fever> 38°C, purulent sputum, antibiotic treatment, or lung atelectasis were present in the four weeks before surgery. A standard staging protocol was adopted. Patients who undergone neoadjuvant treatment (chemotherapy and or radiotherapy) were not included in the present analysis (confounder factor) in order to limit the heterogeneity of the population. Similarly, we have not included in the present study those patients surgically treated with not-anatomical resections (“wedge resections”). Therefore, the surgical procedures consisted of anatomical lung resection (lobectomy or pneumonectomy) with radical nodal dissection in all cases. Finally, adjuvant radiotherapy or chemotherapy was proposed on an individual basis following evidence-based discussions under the care of referring physicians.

### Collected data

Patient’s characteristics, treatment procedures, and short-term outcomes were prospectively collected using a standardized case report form [21]. In particular, serum CRP, albumin and pre-albumin levels were measured in laboratories by nephelometry (as a part of routine pre-surgical laboratory examinations during the study period). Medians were used as cut-off values for statistical analysis. Age, sex, BMI, tobacco consumption, lung function, Karnofsky index were also recorded. Collected pathologic data included histologic type, tumor stage (7^th^ TNM edition [22]) and presence of vascular or lymphatic tumoral emboli. Data on long-term survival were obtained retrospectively through direct telephonic contact with the patient or family. When no clinical follow-up was available, information on vital status was obtained through the municipality of birth of the patient.

### Pathological review

A centralized pathological review of the samples was performed by two expert pathologists. All adenocarcinoma cases have been reclassified independently by two expert pathologists (DD and AML) according to the IASLC/ATS/ERS classification [23] based on predominant architectural pattern. Therefore, adenocarcinomas were graded into three prognostic groups as previously described [24] and the tumour stages were modified in accordance to the 7th edition of the TNM classification [22].

The intra-tumoral density of CD8+ T cells and mature dendritics cells have been analysed in all specimen using immunohistochemistry staining. We have selected the paraffin-embedded tumor block containing the highest density of immune cells and performed immunostaining with the following antibodies CD3 (A0452, Dako cytomation), DC-Lamp (1010E1.01, Dendritics), CD8+ (SP16, Springbioscience) and epithelial antibody (AE1/AE3, Dako cytomation). In order to better identify mature DCs and CD8 T cells within CD3+ T lymphocytes rich area, and tumoral epithelial nests we performed double double labeling CD3/DC-Lamp and CD8/AE1/AE3. Cells were enumerated in the whole section (original magnification ×100), with Calopix software (Tribvn) and results were expressed as an absolute number of positive cells/mm2, as previously described [17]. Quantification was reviewed by two independent observers (JG, MCDN).

CD8+ T cells were found both in stromal area and tumor cell nest. All associations were studied with both values and we found significant associations with both CD8+ T cells location. Because of the potential difficulties in reporting and understanding overlapping data, we decided to report in the paper only the results of immune cells infiltrating tumor cells nests.

### Data analysis

Data processing and analysis were performed with the statistical Software SEM (SILEX Developpment, Mireffleurs, France). Results are expressed as percentage, mean +/−SD for normally distributed and median [interquartile range] for non-normally distributed quantitative variables.

The first step of analyses was the assessment of factors associated with nutritional status (prealbumin level), systemic inflammation (CRP) and tumor immune microenvironment (CD8+ cells and mDC in tumor tissue). Correlations were assessed by the Spearman rank test for continuous variables. Mann-Whitney and Kruskal-Wallis tests were used to perform group comparisons as appropriate. For CD8+ and DC-LAMP+ densities, the “minimum p-value approach” was used to determine the best separation of Kaplan-Meier curves referring to the outcome, with the following cut-offs: CD8: 96 cells/mm^2^ and DC-Lamp: 1.42 cells/mm^2^.

Multivariate analyses (i.e., multilinear regression, including factors significantly associated at univariate analysis) were used to identify factors independently associated with the biomarkers of interest.

Survival analyses were then carried out by Kaplan-Meier method and univariate comparisons were performed using log-rank tests. Risk factors associated with outcomes in univariate analysis with a p-value <0.05 were entered into a multivariate step-by-step Cox model analysis, to identify independent predictors of survival. A p-value <0.05 was considered significant.

## Results

Three-hundred and three patients treated by lobectomy/bilobectomy or pneumonectomy for NSCLC in the study period were analysed. Table 1 summarizes the main preoperative characteristics.

**Table 1:** Clinical, surgical and pathological characteristics and parameters related to nutrition, systemic inflammation and tumoral immune microenvironment in the whole population.

	n (%) or mean ± SD or median [interquartile range]
Men	244 (80.5%)
Age	62 yrs [53–69 yrs]
Smoking history	
Past or present smoking	264 (87.1%)
Smoking cessation at least 2 months before surgery	170 (56.1%)
Cumulative smoking: Pack/Year index	40 [30–50]
Comorbid illnesses	
Alcohol abuse	70 (23.1%)
Diabetes mellitus	35 (11.6%)
Ischaemic heart disease	41 (13.5%)
Stroke	16 (5.3%)
Lower limb atheroma	60 (19.8%)
Respiratory status	
Chronic bronchitis	181 (59.7%)
FEV1 (% predicted)	80.8±18.7
FEV1/FVC (%)	70.9±11.0
ASA I-II/III/IV	13 (4.3%)/187 (61.7%)/100 (33.0%)/3 (1.0%)
Karnofsky 100%/90%/≤80%	97 (32.0%)/110 (36.3%)/96 (31.7%)
Surgical procedures	
Lobectomy/bilobectomy	235 (77.6%)
Pneumonectomy	68 (22.4%)
Histological type	
Squamous cell carcinoma	118 (38.9%)
Adenocarcinoma	137 (45.2%)
Large-cell carcinoma	41 (13.5%)
Others*	7 (2.32%)
Pathological stage	
IA	60 (19.8%)
IB	54 (17.8%)
IIA	47 (15.5%)
IIB	38 (12.5%)
IIIA	88 (29.0%)
IIIB	9 (3.0%)
IV	7 (2.3%)
Vascular emboli	134 (44.2%)
Lymphatic emboli	75 (24.8)%
Body mass index	24.2+4.4 Kg/m^2^
Usual body weight	70 (62–81) Kg
Current body weight	69 (60–80) Kg
CRP	3 (3–17) mg/L
Albumin	45 (40–49) g/L
Prealbumin	285 (220–346) mg/L
Nutritional risk index	107 (99–113)
CD8+ T lymphocytes density	96/mm^2^ (39.7–210.3)
Mature dendritic cells density	1.42/mm^2^ (0.57–3.34)

*these including sarcomatoid carcinomas and adenosquamous carcinomas.

### Factors associated with nutritional status

Prealbumin levels were significantly correlated with other parameters related to nutritional status (body weight, BMI, albumin levels, nutritional risk index), systemic inflammation (inverse relationship with CRP levels) and with several histological features, such as histological type (lower in squamous cell carcinoma), pT parameter, and presence of vascular emboli. Prealbumin levels also correlated positively to mDC density. At multivariate analysis, prealbumin levels were found to be independently associated with CRP levels only (Table 2).

**Table 2:** Associations between the biomarkers related to systemic inflammation, nutrition and tumoral immune microenvironment with clinical and pathological variables.

	Prealbumin	CRP	CD8	mDC
Age	0.835	NS	**0.0062**	**0.0061**
Sex	NS	NS	NS	**0.0011**
Usual body weight	**0.0231**	NS	0.0825	**0.0055**
Actual body weight	**0.0001**	NS	NS	**0.0374**
BMI	**0.0002**	NS	NS	**0.0294**
Smoking status	NS	**0.0055**	NS	**0.0087**
Pack/year	NS	NS	0.0725	0.0813
Alcohol abuse	NS	NS	NS	NS
Chronic bronchitis	NS	NS	NS	0.0621
COPD	NS	NS	NS	**0.0272**
Atheroma	NS	NS	NS	NS
Diabetes	**0.0333**	NS	NS	0.0581
Angor	NS	NS	NS	0.0991
Stroke	NS	**0.0432**	NS	**0.0432**
Karnofsky	NS	NS	NS	NS
ASA	**0.0153**	0.063	NS	**0.0004**
Extent resection	NS	**0.0003**	**0.0081**	0.0947
Pathologic stage	NS	**0.0006**	NS	NS
pT	**0.0011**	**≤0.0001**	NS	0.0551
pN	NS	NS	NS	NS
Histological type	**0.0497**	0.0572	**0.0414**	**0.0008**
Vascular emboli	**0.0192**	0.0773	NS	NS
Lymphatic emboli	NS	NS	NS	NS
Albumin	**≤0.0001**	**≤0.0001**	NS	**<0.0001**
Nutritional risk index	**≤0.0001**	**≤0.0001**	NS	**0.0123**
Prealbumin	NA	**≤0.0001**	NS	**0.0003**
CRP	**≤0.0001**	NA	NS	**≤0.0001**
CD8	NS	NS	NA	**≤0.0001**
mDC	**0.0003**	**≤0.0001**	**0.0001**	NA

P values (univariate analyses) are displayed when <0.1. Significant p values are shown in bold. Independent associations with biomarkers (identified using multivariate analysis) are underlined. Corresponding p-values on multivariate analysis are shown in footnote.

### Factors associated with systemic inflammation

In univariate analysis, CRP levels were correlated to smoking status, extent of resection (higher in pneumonectomy patients), p-stage and pT-parameter, and inversely correlated with albumin levels and nutritional risk index. CRP levels were also inversely correlated with prealbumin levels and mDC density, and these last two associations could be characterized as independent by multilinear regression (Table 2).

### Factors associated with tumor immune microenvironment

**Figure 1:**
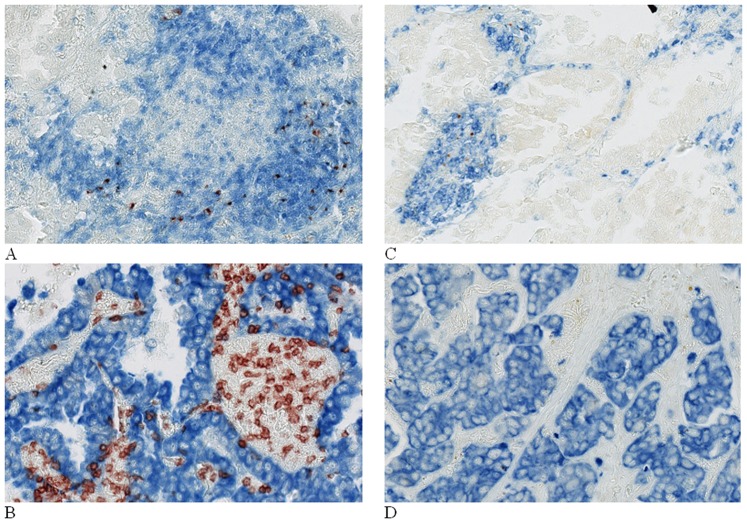
CD8+ and DC-Lamp+ cell densities are heterogeneous in non-small cell carcinoma. Magnification x 100 A) High density of DC-Lamp+ cells (red), these cells are located in CD3+ T-cell rich area (blue). B) Low density of DC-Lamp+ cells. C) High density of CD8+ T cells (red) among pan-cytokeratins+ tumor nests (blue). D) Low density of CD8+ T cells.

The density of CD8+ cells and mature DC is heterogenous among lung carcinoma (Figure 1). The density of CD8+ infiltrating T lymphocytes was found to be correlated with histologic type (lower in squamous cell carcinoma) and extent of resection (lower in pneumonectomy patients) at univariate analysis, as well as with age and density of infiltrating mDC; the association with these last two factors remained significant at multivariate analysis. Moreover, the density of intra-tumoral mDC infiltrating cells was correlated with the main clinical, pathological and laboratory parameters, including age (inverse correlation), sex (higher in women), smoking status (lower in smokers), COPD status (lower in COPD patients), ASA score (lower in higher classes), histology (lower in squamous cell carcinoma), nutritional parameters (body weight, BMI, prealbumin and albumin levels, nutritional risk index), CRP levels (negative correlation), as well as with CD8+ T cells density. Multilinear regression revealed independent associations with presence of COPD, histological type, CRP levels, and CD8+ T cells density (Table 2).

### Long-term survival and prognostic factors

For the whole population, median survival was 52 months; 3, 5, and 7-year overall survivals (OS) were 56.1% (95% C.I: 51.0–62.3), 47.0% (41.3–52.7), and 38.6% (33.0–44.4), respectively. Clinical and pathological factors associated with long-term survival in univariate analysis are shown in Table 3. Of note, chronic bronchitis, Karnofsky index, ASA class, extent of resection, p-stage, pT, and pN parameters as well as the presence of vascular emboli negatively affected survival. IASLC/ATS/ERS classification based on histological grade for adenocarcinoma [23] was found to be an independent prognostic factor. Intermediate grade - lepidic, tubular and papillary predominant adenocarcinoma- had a better prognosis than high grade group - solid predominant, micropapillary predominant and mucinous adenocarcinoma- (p = 0.0521).

**Table 3:** Impact of clinical and pathological variables on survival.

		5-year survival rate	95% C.I.	p
**Age**	<65years	52.0	44.3–59.54	0.0612
	>65 years	41.2	32.9–49.9	
**Sex**	Men	45.9	39.8–52.1	0.4222
	Women	50.4	35.8–64.8	
**BMI**	<18.5	41.7	24.5–61.2	0.0980
	18.5–24.9	43.7	35.9–51.9	
	≥25	53.8	44.8–62.6	
**Smoking status**	Current and former	46.9	41.0–52.8	0.3178
	Never	56.0	31.0–78.2	
	>40 pack/year	44.1	35.4–53.1	02512
	≤40 pack/year	50.8	42.9–58.7	
**Alcohol abuse**	Yes	43.1	32.8–54.0	0.6511
	No	48.5	41.8–55.3	
**Chronic bronchitis**	Yes	42.2	35.5–49.2	**0.0198**
	No	56.6	46.5–66.2	
**COPD**	Yes	40.8	31.65–50.68	0.2210
	No	50.1	42.1–58.1	
**Atheroma**	Yes	43.5	30.6–57.3	0.2850
	No	50.4	42.9–57.9	
**Diabetes**	Yes	35.5	22.1–51.6	0.4333
	No	47.6	41.2–54.0	
**Angor**	Yes	46.0	29.9–63.0	0.4333
	No	49.2	41.7–56.7	
**Stroke**	Yes	23.1	8.2–50.3	0.0666
	No	49.4	42.2–56.5	
**Karnofsky**	100	68.2	53.4–80.1	**0.0250**
	≤90	43.7	34.4–53.5	
**ASA**	I-II	51.6	44.4–58.7	**0.0198**
	III-IV	38.4	29.4–48.3	
**Extent resection**	Lobectomy/bilobectomy	53.3	46.8–59.8	**<0.0001**
	Pneumonectomy	24.4	15.6–36.0	
**Pathologic stage**	I	67.2	58.6–74.8	**<0.0001**
	II	37.2	26.3–49.5	
	III-IV	28.6	20.6–38.2	
**pT**	T1	65.3	53.7–75.5	**<0.0001**
	T2	51.0	42.5–59.3	
	T3	26.6	16.9–39.2	
	T4	26.4	13.4–44.8	
**pN**	N0	58.4	50.7–65.8	**<0.0001**
	N1	38.8	27.6–51.3	
	N2	26.2	16.9–38.1	
**Histological type**	Non-squamous	52.8	43.8–61.7	0.2723
	Squamous	46.8	37.9–55.8	
**Vascular emboli**	Yes	38.2	30.2–47.0	**0.0310**
	No	54.3	46.6–61.7	
**Lymphatic emboli**	Yes	40.1	29.7–51.4	0.1278
	No	49.8	43.1–56.4	

Whole population. Univariate analyses.

**Figure 2:**
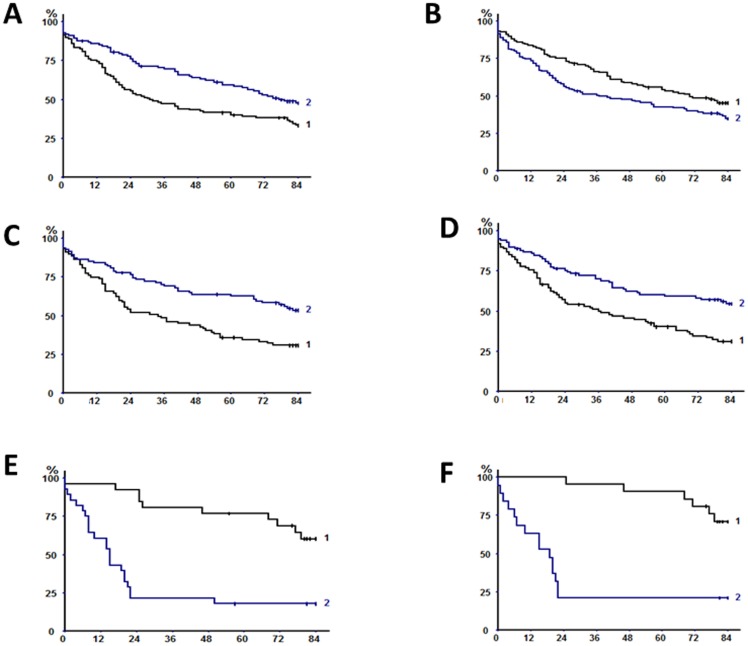
Kaplan-Meyer analysis of overall survivals. A) according to the prealbumin levels, in the whole population. B) according to CRP levels, in the whole population. C) according to CD8+ T cells density, in the whole population. D) according to mDC density, in the whole population, E) according to combination of CRP, prealbumin, and CD8 levels (≤3,>285 and>96 [1] vs>3, <285 et <96 [2]), in the whole population. Intermediate groups are not displayed. F) according to combination of CRP, prealbumin, and CD8 levels (≤3,>285 and>96 [1] vs>3, <285 et <96 [2]), in Stage I-II disease. Intermediate groups are not displayed.

Table 4 summarizes univariate associations between survival and biomarkers of nutritional status, systemic inflammation and tumoral immune microenvironment. In particular, prealbumin levels may predict the long-term outcome (p = 0.0087; 5-year survival rates of 39.9% and 58.3% in patients with levels below and superior to median value, respectively, Figure 2A). In agreement with our previous report [14], systemic inflammation also influenced the long-term survival, with undetectable (≤3 mg/L) CRP levels being associated to more favorable outcome (p = 0.0372; 5-year survival rate of 54.1% vs 42.6%, Figure 2B). Similarly, the tumoral immune microenvironment was proven to be a strong predictor of long-term outcome; indeed, high densities (superior to median value) of either CD8+ T lymphocytes or mDC were found to be related with a significantly better prognosis (5-year survival rates of 35.7% and 63.3% and of 40.2% and 59.2% in patients with low and high density of CD8+ T cells, and of mDC, respectively, p = 0.0312 and p = 0.0015, Figures 2C-D).

**Table 4:** Impact of parameters related to nutrition, systemic inflammation and tumoral immune microenvironment on survival.

		5-year survival rate	95% C.I.	p
**BMI**	<18.5	41.7	24.5–61.2	0.0980
	18.5–24.9	43.7	35.9–51.9	
	≥25	53.8	44.8–62.6	
**Usual body weight**	≤70	45.4	37.1–54.0	0.2810
	>70	51.0	42.9–59.0	
**Actual body weight**	≤69	41.9	34.2–50.0	0.0564
	>69	52.8	44.5–61.2	
**Albumin**	>45 g/L	54.5	44.9–63.8	**0.0420**
	≤45 g/L	44.3	36.9–52.0	
**Buzby’s risk index**	≤83.5	28.6	11.7–54.6	0.2610
	83.6–97.5	54.5	37.2–70.8	
	>97.5	51.0	42.8–59.1	
**Prealbumin**	>285 mg/L	58.3	49.0–67.1	**0.0087**
	≤285 mg/L	39.9	31.7–48.6	
**CRP**	>3 mg/L	42.6	34.8–50.8	**0.0370**
	≤3 mg/L	54.1	45.6–62.3	
**CD8**	≤96/mm2	35.7	26.4–46.2	**0.0028**
	>96/mm2	63.3	53.1–72.4	
**mDC**	≤1.42/mm2	40.2	31.0–50.2	**0.0015**
	>1.42/mm2	59.2	50.3–69.6	

Whole population. Univariate analysis.

We defined subgroups of patients based on biomarkers levels of systemic inflammation, nutritional status and tumoral immune microenvironment. The resulting categorization was highly discriminant in terms of survival (p<0.0001): patients with the following “pattern” (CRP≤3 mg/L, prealbumin>285 mg/L and>96/mm^2^ CD8+ cell density) had a better 5-year survival rate (80% versus 18%, respectively; figure 2E) when compared with those patients with an opposite pattern (CRP levels>3 mg/L, prealbumin level≤285 mg/L and CD8+ cell density ≤96/mm^2^). Patients in the other subgroups showed intermediate prognosis (Table 5).

**Table 5:** Impact on survival of association of parameters related to nutrition, systemic inflammation and tumoral immune microenvironment. Whole population.

	5-year survival rate	95% C.I.	p
CRP≤3 and DC>1.42	63.2	49.8–74.7	0.0037
CRP>3 and DC≤1.42	35.8	25.1–48.2	
CRP≤3 and DC≤1.42	50.4	34.0–66.7	
CRP>3 and DC>1.42	59.9	49.5–69.4	
CRP≤3 and CD8>96	70.0	57.2–83.9	0.0005
CRP>3 and CD8≤96	26.2	15.7–40.4	
CRP≤3 and CD8≤96	50.0	34.8–65.1	
CRP>3 and CD8>96	56.6	42.9–69.3	
Prealbumin>285 and DC>1.42	75.0	63.0–87.2	0.0009
Prealbumin≤285 and DC≤1.42	36.4	24.3–50.5	
Prealbumin>285 and DC≤1.42	56.8	37.9–74.0	
Prealbumin≤285 and DC>1.42	42.4	27.2–59.2	
Prealbumin>285 and CD8>96	73.0	57.0–84.6	
Prealbumin≤285 and CD8≤96	18.4	9.2–33.4	
Prealbumin>285 and CD8≤96	70.4	51.5–84.1	
Prealbumin≤285 and CD8>96	54.0	38.7–68.5	
CRP≤3, prealbumin>285 and CD8>96	80.0	60.9–91.1	<0.0001
CRP>3, prealbumin≤285 and CD8≤96	18.0	7.9–35.6	
CRP>3, prealbumin≤285 and CD8>96	55.2	37.5–71.6	
CRP<3, prealbumin≤285 and CD8>96	44.4	18.9–73.3	
CRP>3, prealbumin>285 and CD8>96	63.6	35.4–84.8	
CRP≤3, prealbumin>285 and CD8≤96	72.2	49.1–87.5	
CRP≤3, prealbumin≤285 and CD8≤96	20.0	5.7–51.0	
CRP>3, prealbumin>285 and CD8≤96	66.7	35.4–87.9	

Cox models were then built. Since significant correlations were found between CRP and prealbumin levels on the one hand, and CD8+ T cells and mDC densities on the other hand, these variables were mutually exclusive. Thus, the first model included chronic bronchitis, Karnowski PS, ASA class, extent of resection, stage of disease, presence of vascular emboli, prealbumin levels and CD8+ T cells density. Prealbumin levels, CD8+ T cells density, and disease stage were finally identified as independent prognostic markers (Table 6). The second model, in which CRP levels replaced prealbumin levels, identified stage and CD8+ T cells density as independent prognostic factors.

**Table 6:** Multivariate analysis of prognostic factors of survival in the whole population.

**Model 1**	**p**		**RR**	**95% C.I.**
Prealbumin	0.0055	**≤**285 mg/L	1	
		>285 mg/L	0.34	0.16–0.73
CD8	0.0160	≤96/mm2	1	
		>96/mm2	0.37	0.16–0.83
Stage	0.0370	I	1	
		II	1.73	1.03–2.89
		III-IV	2.99	1.07–8.37
**Model 2**	**p**		**RR**	**95% C.I.**
CD8	0.0120	≤96/mm2	1	
		>96/mm2	0.35	0.12–0.87
Stage	0.0246	I	1	
		II	1.82	1.02–2.97
		III-IV	3.04	1.17–8.27

When stage I-II disease were considered alone, the prognostic impact of factors related to poor nutrition, systemic inflammation and tumoral immune cell infiltration was even more remarkable (Table 7); in detail, the prealbumin and CRP levels, as well as intra-tumoral CD8+ T cells and mDC density strongly predicted the long-term outcome (p = 0.0008, p = 0.0049, p = 0.0004, and p = 0.0050, respectively). Taken together, their prognostic value was very high, allowing a powerful separation of patients groups: 5-year survival rate was 86.4% in patients with CRP≤3 mg/L, prealbumin levels>285 mg/L, and CD8+ T cells density>96/mm^2^ versus 21.1% in patients with CRP>3 mg/L, prealbumin levels≤285 mg/L, and CD8+ T cells density ≤96/mm^2^ (Figure 2F). As for the whole population, two Cox models were generated for stage I-II disease, one including prealbumin levels and CD8+ T cells density together with the other significant clinical and pathological variables, and the other including CRP levels instead of prealbumine levels. In both models (Table 8), CD8+ T cells density and either prealbumin or CRP levels (depending on which of these two variables was entered in the statistical model) could be identified as independent prognostic factors.

**Table 7:** Impact of parameters related to nutrition, systemic inflammation and tumoral immune microenvironment on survival in patients with stage I-II disease.

	5-year survival rate	95% C.I.	p
**Prealbumin**>285 mg/L	70.0	59.0–79.1	0.0008
**Prealbumin**≤285 mg/L	44.4	33.9–55.3	
**CRP**≤3 mg/L	64.8	54.9–73.6	0.0049
**CRP**>3 mg/L	48.4	38.1–58.7	
**CD8**>96/mm2	71.6	59.9–81.0	0.0004
**CD8**≤96/mm2	42.4	30.6–55.1	
**mDC**>1.42/mm2	68.9	56.8–78.9	0.0050
**mDC**≤1.42/mm2	48.6	36.7–60.5	
**CRP≤3, prealbumin>285 and CD8>96**	86.4	66.7–95.2	0.0063
**CRP>3, prealbumin ≤285 and CD8≤96**	21.1	8.5–43.3	
**CRP≤3, prealbumin>285 and DC>1.42**	85.3	67.7–94.1	0.0004
**CRP>3, prealbumin≤285 and DC≤1.42**	44.4	27.6–63.0	

Univariate analysis.

**Table 8:** Multivariate analysis of prognostic factors of survival in the stage I-II disease.

**Model 1**	**p**		**RR**	**95% C.I.**
Prealbumin	0.0210	≤285 mg/L	1	
		>285 mg/L	0.41	0.23–0.73
CD8	0.0162	≤96/mm2	1	
		>96/mm2	0.41	0.23–0.71
**Model 2**	**p**		**RR**	**95% C.I.**
CRP	0.0240	≤3	1	
		>3	1.78	1.08–2.93
CD8	0.0041	≤96/mm2	1	
		>96/mm2	0.49	0.30–0.80
Stage	0.0377	I	1	
		II	1.87	1.12–3.12

## Discussion

In this large cohort of consecutive patients it was found that, in resected NSCLC patients, biomarkers related to systemic inflammation, nutritional status, and tumoral immune microenvironment are interrelated and these represent major determinants of long-term outcome that, when taken into account together, allow highly robust discrimination of groups of patients with different prognosis.

### Role of systemic inflammation

CRP is secreted by hepatocytes following stimulation by circulating pro-inflammatory cytokines, in particular IL-1, TNF-α, and mainly IL-6 [25]. Experimental studies [26]–[28] have suggested that NSCLC cells are able to release IL-6 and TNF-α. In spite of this, the exact role of systemic inflammation and tumor burden in determining progression and outcome is still controversial. This relationship is even more questionable in “pre-clinical disease”: a study on a large cohort showed that increased CRP levels in cancer-free subjects were associated with a higher risk of lung cancer occurrence [12]. This finding has been recently confirmed by a nested case-control study [11]: among 77 evaluated inflammatory biomarkers, 11 were found to be associated with an increased risk of developing lung cancer, even after adjustment for smoking. Among these 11 markers, CRP was the most robust predictor of lung cancer risk. [11]. Moreover, increased baseline CRP levels were associated with early death after diagnosis of any cancer in patients without metastatic disease at diagnosis [12]. These findings strongly suggest a possible role of pre-existing systemic inflammation in determining the occurrence and prognosis of lung cancer. In our population, CRP levels were an independent prognostic factor in stage I-II disease only. Higher CRP levels were associated to higher pT parameter and (albeit non-significant) higher occurrence of vascular emboli, an important determinant of cancer progression and spread. Similarly, systemic inflammation has been reported to be an independent negative prognostic marker in patients with advanced non-small cell lung cancer [29].

### Nutritional status

In the present study, we found that CRP levels were strongly and independently correlated (in an inverse manner) with prealbumin levels. Prealbumin levels were in turn correlated with pT parameter, vascular embols, and, as for CRP levels, intra-tumoral density of mDC. Such correlations underline the complex interplay between systemic inflammation, malnutrition, and tumoral immune microenvironment; we may theorize that malnutrition is the first cause of immunodeficiency [30], and in lung cancer patients this could result in poor infiltration of anti-tumoral immune cells. Therefore, this would explain the strong negative impact of low prealbumin levels on long-term survival. Furthermore, inflammatory status (pre-existent or concomitant with lung cancer) with subsequent increased energy consumption might contribute to malnutrition.

### Tumor immune microenvironment

As already demonstrated [15], [17], high intra-tumoral densities of mDC and CD8+ T lymphocytes were associated with improved outcome. Interestingly, mDC density in lung cancer reflects the immune response organization within tertiary lymphoid structures (TLS) adjacent to the tumor nests, where CD8+ T cells are supposed to be educated for an efficient antitumor immune response [17], [28].

In our study, intratumoral mDC density was associated with relevant clinical and biological parameters including not only nutritional ones but also (in an inverse manner) those associated with systemic (CRP levels) and local (smoking, COPD) inflammation. A very strong correlation existed between mDC and CD8+ T cell densities, underlining the link between both cell types [17]. Determinants of the immune contexture for a given tumor are still not well understood. Specifically, it is not known if the tumor immune microenvironment is shaped by tumor cells themselves or by patients’ underlying characteristics. In this context, Kikuchi and coll. [31] have explored the role of Human Leukocyte Antigen (HLA) class I that displays a repertoire of endogenously processed peptides to CD8 (+) T lymphocytes. The authors observed that the down-regulation of HLA class I expression in NSCLC is a marker of poor prognosis, and this may play a critical role in immune surveillance of patients with NSCLC.

Interestingly we have observed for the first time to our knowledge, that tumor immune microenvironment is linked to nutritional status and systemic inflammation as reflected by the correlation between mDC density and several conditions and clinical features (such as stroke, COPD, usual body weight, CRP and prealbumin levels, etc.). Overall, our results suggest that preexisting systemic inflammation/poor nutritional status could impact the intra-tumoral immune contexture and the patient survival.

### Role of biomarkers as predictors of survival

When associating mDC or CD8+ T cell densities with either CRP or prealbumin levels, we could identify subgroups of patients with significantly different long-term outcomes.

The best discrimination was achieved when taking into account simultaneously biomarkers related to inflammation with nutritional status and intra-tumoral immune infiltration. With this model, the differences in survival were remarkable when comparing, in the whole population as in stage I-II disease, patients with high CD8+ T cells density, low CRP levels and high prealbumin levels to those with low CD8+ T cells density, high CRP levels and low prealbumin levels. Interestingly, groups with intermediate biological characteristics had intermediate long-term outcomes.

### Limitations of the study

Our study suffers from the common bias of investigations on surgical registries as well as the retrospective nature and a certain degree of heterogeneity of the sample (uncontrolled cohort of multiple stages and therapies). Missing data led us to exclude some potentially eligible patients, however this limitation concerned only a minority of patients, and is therefore unlikely to have affected the results. Such limitations should be kept firmly in mind by the readers when considering the clinical implications deriving from the present analysis.

### Perspectives and Conclusions

According to our data, systemic inflammation and poor nutritional status seems to be associated with poor outcome in lung cancer patients. Similarly, intra-tumoral immune cells characteristics appear to significantly influence the long-term outcome in such patients. The interplay between tumor, immunologic microenvironment, inflammation, and nutrition is complex, as underlined by our results, and therefore remains to be fully understood, but is likely of paramount importance for the development of novel prognostic markers and therapeutic strategies.
